# Transgender health in medical education

**DOI:** 10.2471/BLT.19.249086

**Published:** 2021-01-21

**Authors:** Tommy Hana, Kat Butler, L Trevor Young, Gerardo Zamora, June Sing Hong Lam

**Affiliations:** a Faculty of Medicine, University of Toronto, Toronto, Ontario, Canada.; b Gender, Equity and Human Rights Team, World Health Organization, Geneva, Switzerland.; c Centre for Addiction and Mental Health, 60 White Squirrel Way, Toronto, M6J 1H4, Ontario, Canada.

## Abstract

Transforming our world the 2030 agenda for sustainable development is working towards a world that reflects equity, with universal respect for human dignity, pledging to leave no one behind. However, transgender and gender-diverse individuals experience significant health inequities, including negative health outcomes and multiple barriers to accessing care. In this article, we first highlight the health inequities that transgender and gender-diverse people face globally. We describe important aspects of transgender and gender-diverse health care, including the design and provision of health services, epidemiological considerations, transition-related care, changes in transition-related goals, cultural considerations, and political and legal issues. We then review the existing global literature on incorporating transgender health into medical curricula. We make a case for prioritizing improved education in medical schools on the specific health needs of transgender and gender-diverse people as part of addressing global health inequities in care. Our recommendations for comprehensive education on transgender health include cultural humility and anti-oppression training; involvement of transgender and gender-diverse community members; integration of transgender and gender-diverse health into curricula; practice-focused and in situ training; staff development in medical schools; and improving access to careers in medicine for transgender and gender-diverse people.

## Résumé

Transformer notre monde: le Programme de développement durable à l'horizon 2030 aspire à un monde où règne l'équité, le respect universel de la dignité humaine, et s'engage à ce que personne ne soit oublié. Pourtant, les individus transgenres et de genre variant subissent de profondes inégalités sur le plan sanitaire, qui affectent leur état de santé et compliquent grandement l'accès aux soins. Dans cet article, nous commençons par souligner les inégalités sanitaires auxquelles sont confrontés les individus transgenres et de genre variant à travers le monde. Nous évoquons d'importants aspects à prendre en compte pour les soins de santé aux personnes transgenres et de genre variant, parmi lesquels la conception et la fourniture de services de santé, les considérations d'ordre épidémiologique, les soins liés à la transition, l'évolution des objectifs liés à la transition, les facteurs culturels, ainsi que les enjeux politiques et juridiques. Nous examinons ensuite la littérature internationale consacrée à l'intégration de la santé transgenre dans les programmes d'enseignement médical. Nous soutenons que, dans le cadre de la lutte contre les inégalités sanitaires au niveau mondial, il faut privilégier une amélioration de l'enseignement dans les écoles de médecine afin de sensibiliser aux besoins spécifiques des individus transgenres et de genre variant en matière de santé. Nous avons formulé plusieurs recommandations en vue d'instaurer un enseignement qui tient compte de la santé des personnes transgenres: apprentissage anti-oppressif intégrant la notion d'humilité culturelle; implication de membres de la communauté transgenre et de genre variant; ajout de la santé transgenre et de genre variant dans les programmes de cours; formation centrée sur la pratique, dispensée sur le terrain; développement du personnel des écoles de médecine; et enfin, amélioration de l'accès aux carrières médicales pour les individus transgenres et de genre variant.

## Resumen

Transforming our world: the 2030 agenda for sustainable development (Transformar de nuestro mundo: la Agenda 2030 para el desarrollo sostenible) trabaja por un mundo que refleje la igualdad, con respeto universal por la dignidad humana, comprometiéndose a no dejar a nadie atrás. Sin embargo, las personas transgénero y con género diverso experimentan importantes desigualdades en materia de salud, incluidos resultados sanitarios negativos y múltiples obstáculos para acceder a la atención sanitaria. En este artículo, destacamos, en primer lugar, las desigualdades sanitarias a las que se enfrentan las personas transgénero y de género diverso en todo el mundo. Describimos aspectos importantes de la atención sanitaria a las personas transgénero y con género diverso, como el diseño y la prestación de servicios sanitarios, las consideraciones epidemiológicas, la atención relacionada con la transición, los cambios en los objetivos relacionados con la transición, las consideraciones culturales y las cuestiones políticas y jurídicas. A continuación, revisamos la documentación global existente sobre la incorporación de la salud transgénero en los planes de estudio de medicina. Defendemos la necesidad de dar prioridad a la mejora de la formación en las facultades de medicina sobre las necesidades sanitarias específicas de las personas transgénero y con género diverso como parte del tratamiento de las desigualdades sanitarias globales en la atención sanitaria. Nuestras recomendaciones para una educación integral sobre la salud de las personas transgénero incluyen la formación en humildad cultural y lucha contra la opresión; la participación de los miembros de la comunidad transgénero y con género diverso; la integración de la salud de las personas transgénero y con género diverso en los planes de estudio; la formación centrada en la práctica e in situ; el desarrollo del personal en las facultades de medicina; y la mejora del acceso a las carreras de medicina para las personas transgénero y con género diverso.

## ملخص

تحويل عالمنا جدول أعمال عام 2030 للتنمية المستدامة: يسعى نحو عالم تتحقق فيه العدالة، مع الاحترام العالمي لكرامة الإنسان، والتعهد بعدم إهمال أي شخص. ومع ذلك، يعاني الأفراد المتحولون جنسياً وأصحاب التنوع الجنسي من تفاوتات صحية كبيرة، بما يشمل النتائج الصحية السلبية والعوائق المتعددة أمام الحصول على الرعاية. نقوم أولاً في هذه المقالة بالتركيز على التفاوتات الصحية التي يواجهها المتحولون جنسياً وأصحاب التنوع الجنسي على مستوى العالم. نحن نصف الجوانب المهمة للرعاية الصحية للمتحولين جنسياً وأصحاب التنوع الجنسي، بما في ذلك تصميم وتقديم الخدمات الصحية، والاعتبارات الوبائية، والرعاية المتعلقة بالتحول، والتغييرات في الأهداف المتعلقة بالتحول، والاعتبارات الثقافية، والمسائل السياسية والقانونية. ثم نراجع الثوابت العالمية الموجودة بخصوص دمج صحة المتحولين جنسيًا في المناهج الطبية. نحن نسعى لإعطاء الأولوية للتعليم المحسن في كليات الطب حول الاحتياجات الصحية المحددة للأشخاص المتحولين جنسياً وأصحاب التنوع الجنسي كجزء من التعامل مع التفاوتات الصحية العالمية في الرعاية. إن توصياتنا للتعليم الشامل حول صحة المتحولين جنسياً تشمل التدريب لمواجهة التحرش الثقافي ومكافحة الاضطهاد؛ وإشراك أعضاء المجتمع المتحولين جنسياً وأصحاب التنوع الجنسي؛ ودمج صحة المتحولين جنسياً وأصحاب التنوع الجنسي في المناهج الدراسية؛ والتدريب بالتركيز على الممارسة وفي الموقع؛ وتطوير فرق العمل في كليات الطب؛ وتحسين الحصول على وظائف طبية للأشخاص المتحولين جنسياً وأصحاب التنوع الجنسي.

## 摘要

《改变我们的世界：2030 年可持续发展议程》致力于建立一个体现公平、普遍重视人类尊严的世界，保证不让任何一个人掉队。但是，跨性别者和性别多样化个体遭遇了严重的健康不平等问题，包括负面的健康结果以及在获得护理方面面临的多重障碍。在本文中，我们首先强调了全球跨性别者和性别多样化人群所面临的健康不平等问题。描述了跨性别者和性别多样化群体医疗保健服务的各个重要方面，包括卫生服务的设计和供应、流行病学考量、变性相关护理、变性相关目标变化、文化考量以及政治和法律问题。然后，我们回顾了有关将跨性别者健康问题纳入医学课程的全球现有文献。作为解决全球医疗卫生不平等问题的一部分，我们充分说明了优先要求医学院就跨性别者和性别多样化人群的特殊健康需求问题加强教育的原因。对于如何就跨性别者健康问题全面开展教育，我们的建议包括：提供宣扬谦逊精神和反压迫的培训；让跨性别者和性别多样化人群的社区成员参与；将跨性别者和性别多样化人群的健康问题纳入课程；提供注重实践的现场培训；在医学院加强医护人员的培训；以及为跨性别者和性别多样化人群提供更多进入医疗界的就业机会。

## Резюме

Документ Преобразование нашего мира: повестка дня в области устойчивого развития на период до 2030 годанаправлен на построение мира, который отражает равенство, всеобщее уважение человеческого достоинства и обязательство принятия во внимание интересов всех и каждого. Однако трансгендеры и люди с разнообразной гендерной самоидентификацией сталкиваются со значительным неравенством в отношении здоровья, включая негативные последствия для здоровья и множественные препятствия для доступа к медицинской помощи. В этой статье прежде всего подчеркивается несправедливость в отношении здоровья, с которой трансгендеры и люди с разнообразной гендерной самоидентификацией сталкиваются в глобальном масштабе. Авторы описывают важные аспекты медико-санитарной помощи трансгендерам и людям с разнообразной гендерной самоидентификацией, включая разработку и предоставление услуг здравоохранения, эпидемиологические соображения, медицинскую помощь в процессе смены пола, изменения в целях, связанных с процессом смены пола, культурные аспекты, а также политические и юридические вопросы. Затем следует обзор существующей мировой литературы по включению здоровья трансгендеров в медицинские учебные программы. Авторы приводят доводы в пользу придания приоритетной значимости повышению качества образования в медицинских институтах в отношении особых медицинских потребностей трансгендеров и людей с разнообразной гендерной самоидентификацией в рамках решения проблемы глобального неравенства в отношении здоровья при оказании медицинской помощи. Рекомендации по всестороннему обучению в вопросах здоровья трансгендеров включают: культурное принятие и обучение по принципам отсутствия притеснения; вовлечение в этот процесс трансгендеров и представителей групп людей с разнообразной гендерной самоидентификацией; включение вопросов здоровья трансгендеров и людей с разнообразной гендерной самоидентификацией в учебные программы; практическое обучение и обучение на месте; повышение квалификации сотрудников медицинских институтов; улучшение доступа к возможностям карьерного роста в медицинской сфере для трансгендеров и людей с разнообразной гендерной самоидентификацией.

## Introduction

Transgender people are individuals with a gender identity different from the sex assigned to them at birth. The term transgender can be used to encapsulate various gender identities, including gender-diverse individuals who identify outside the socially constructed gender binary of male and female. While these concepts are becoming increasingly familiar in some countries, other terms may be used to describe people who inhabit transgender, gender-diverse and non-binary gender identities. Some countries recognize more than two genders both in law and cultural traditions and may provide legal protection for these groups due to their cultural, traditional or religious significance. Specific indigenous terms for transgender and gender-diverse people include Hijra (India), meti (Nepal), skesana (South Africa), waria (Indonesia), travesti (Argentina, Brazil), muxé (Mexico), fa’afafine (Samoa), fakaleiti (Tonga) and Two-Spirit (North American Indigenous coalition).^1^

Studies from different countries have estimated the prevalence of transgender and gender-diverse individuals to be between 0.5% and 1.2% of the population.^2^ Transgender and gender-diverse people are disproportionately affected by human immunodeficiency virus (HIV) infection, depression, anxiety and risk of suicide, exacerbated by experiences of oppression, discrimination and violence.^2^ The health needs of transgender and gender-diverse people have frequently been pushed to the margins or ignored in health agendas.^3^

For many years transgender and gender-diverse identities were categorized as mental disorders by mental health clinicians. The 11th revision of the *International statistical classification of diseases and related health problems* (ICD-11) reframed gender identity-related health issues by replacing outdated diagnostic categories with the diagnosis of gender incongruence, which describes the experience that one’s gender is different from the sex assigned at birth. Gender incongruence was moved from the Mental and Behavioural Disorders chapter to the new Conditions Related to Sexual Health chapter in 2018. This reclassification helps to clarify that transgender and gender-diverse identities are not inherently pathological, while still facilitating appropriate health-care services for and access to gender-affirming care. This diagnosis in the ICD-11 also acknowledges the important links between gender identity and sexual behaviour, exposure to violence, sexually transmitted infections and other health-related issues.^4^

*Transforming our world: the 2030 agenda for sustainable development* is working towards a world that reflects equity with universal respect for human dignity, pledging to leave no one behind.^5^ The respect and protection of internationally recognized human rights, such as the right to the highest attainable standard of health and the right to non-discrimination, require that all people have access to high-quality and affordable health services, including services related to gender-affirming care and conditions relating to sexual health.^6^^,^^7^

In health-care systems around the world, there are gaps both in addressing the specific health needs of transgender and gender-diverse patients and in providing general health care to these patients with acceptable sensitivity, dignity and respect. In some countries, these gaps are exacerbated by legal systems that criminalize the fundamental identity of transgender and gender-diverse people and by a lack of gender-affirming legislation, including anti-discrimination protections.^8^ Other contributors to this gap are prejudice, inadequate training, and conscious and unconscious bias among health-care workers, as well as cis-normative service models that presume all patients, learners and clinicians are not transgender or gender diverse.^8^

In this article, we highlight the health inequities that transgender and gender-diverse people face globally and make a case for prioritizing improved transgender health education in medical school curricula as part of addressing the gap in care.

## Overview of transgender health

### Service design and provision

Gender-affirming care can broadly include social, psychological, behavioural and medical interventions designed to support and affirm an individual’s gender identity.^9^ This care could range from gender-affirming primary health care and psychotherapy to hormone therapy and transition-related surgery.^10^ Gender-affirming care should be delivered with a person-centred, equity- and rights-based, non-judgemental, safe and empowering approach. Gender-affirming care requires clinicians to be competent in caring for transgender and gender-diverse people, aware of the general and specific health-care needs of this group, and to behave without discrimination.^11^

Transgender and gender-diverse people face many barriers to accessing gender-affirming care. First, they face oppression, marginalization, discrimination and violence that affect both their social determinants of health and access to health care. These factors lead to high rates of unemployment, poverty, housing instability, lack of educational opportunities, lack of social support systems and other social challenges, all of which make accessing care challenging.^2^ In instances where transgender and gender-diverse people are able to access care, many individuals fear that clinicians will act in discriminatory ways, be unsupportive or provide inadequate care. These fears are often supported by individuals’ experiences of the health-care system.^12^^–^^16^ Clinicians who are poorly educated or biased about gender diversity may be deliberately or accidentally discriminatory in their words or behaviour. Such clinicians contribute to and perpetuate institutional and systemic discrimination, which often leads to denial of the existence of transgender and gender-diverse people, with significant negative impacts on their health outcomes and on access to care consistent with that received by the general population.^17^

Seeking care can mean risking harm for transgender and gender-diverse individuals,^18^ as many countries do not have legal protections for them.^19^ Non-inclusive policies in institutions, including hospitals and clinics, can perpetuate transphobia and avoidance of health care for these individuals.^17^ Such policies include a lack of diverse gender identities on hospital data collection forms (such as registration forms) and electronic records; rooms or wards designated by assigned sex; and lack of access to appropriate gender-inclusive bathrooms. Improving access to care for transgender and gender-diverse people therefore requires a multipronged approach including education of clinicians in gender-affirming care; policy change in health-care institutions; and systemic advocacy to address the social determinants of health. As leaders in health care, physicians need to be trained in how to recognize and address these issues if they are to truly provide equitable care for transgender and gender-diverse people.

### Epidemiological considerations

Beyond challenges in accessing primary health care, transgender and gender-diverse individuals also face barriers to addressing their specific health-care needs. These barriers translate into transgender and gender-diverse people having a higher prevalence of mental health disorders, including depression and anxiety disorders and suicidality, related to various levels of oppression and marginalization.^20^^–^^23^ The higher prevalence of these illnesses is often understood in the context of the minority stress model, which proposes that disproportionately high rates of negative health outcomes are at least in part secondary to the stigma and discrimination one experiences due to an aspect of one’s identity.^22^ One example is the elevated risk of substance use, reported in several studies of transgender and gender-diverse people, which is commonly conceptualized as a response to minority stress.^2^^,^^20^^–^^23^ Individuals may also cope with minority stress through higher-risk sexual practices, which may contribute to increased rates of sexually transmitted infections.^2^^,^^20^^,^^21^ For instance, transgender and gender-diverse people assigned male at birth are disproportionately affected by sexually transmitted infections, such as HIV.^2^^,^^20^

Access to gender-affirming hormone therapy, while crucial for the well-being of some transgender and gender-diverse people, can increase their risk of metabolic, cardiovascular and other health outcomes that require regular screening and possibly treatment.^24^^,^^25^ Transition-related surgery, like all surgical procedures, may lead to complications, and clinicians need to be aware of these to provide appropriate follow-up care.^26^ Given the specific health needs of transgender and gender-diverse people, high-quality care for them requires clinicians to have basic knowledge about gender diversity as well as dedicated education on both the general and specific health-care needs of this group within their cultural context.

### Transition-related care

Affirming care includes supporting transgender and gender-diverse people in their transition-related goals. Transition often broadly includes social transition and medical transition. Social transition includes changing one’s gender expression to more closely match one’s gender identity and can include changing one’s name, pronouns, clothing, hair style, make-up, use of accessories and other aspects of social transition. Social transition, including for children and young people, has been found to be associated with improved mental health outcomes and quality of life measures.^27^ Clinicians can support social transition by being aware of diverse gender identities and aspects of transitioning; offering validation, flexibility and non-judgemental support regarding transition goals; knowing and providing resources on social transitioning (for example, where and how to change one’s gender marker on government-issued identification documents); and facilitating discussions with family members and other loved ones on diverse gender identities and transition-related goals if this is consistent with the patient’s goals.^27^

Medical transition involves medical interventions, including potentially hormone therapy and surgeries, to affirm one’s gender identity. Not every transgender or gender-diverse individual wants to medically transition, and individuals may choose certain medical transition options and not others. Many countries do not offer access to medical transition, and even fewer countries will cover the associated costs, so transgender and gender-diverse individuals often travel internationally for transition-related surgery and purchase hormone therapy from other countries, which can increase risk and health complications.^2^^,^^28^ Medical transition for those with these goals has been associated with significantly improved mental health outcomes and quality of life measures, including reduced risk of suicide.^27^^,^^29^^,^^30^ Therefore, gender-affirming care requires educating clinicians on the importance of assessing for and supporting transition-related goals. Specific training around how to prescribe hormone therapy and how to perform certain surgical or procedural techniques is also required to increase physicians’ capacity for gender-affirming care. Some countries require a diagnosis of Gender Incongruence (as per the ICD-11) or Gender Dysphoria (as per the *Diagnostic and statistical manual of mental disorders, 5th edition*) to support access to medical transitioning. Clinicians should therefore also be offered training in diagnosing these conditions, consistent with the specific national context.

### Cultural considerations

Although transgender and gender-diverse people are present in cultures throughout the world, their existence conflicts with the normative sex and gender binary system and they face discrimination in most societies. Perceptions of transgender and gender-diverse individuals are impacted by culture, religion, and historical contexts. Colonial governments have perpetuated a gender binary system as a form of colonial oppression in places where gender diversity has been part of the local cultural context.^31^^,^^32^ Such factors have impacted what is considered the accepted presentation of gender identity based on socially constructed gender norms. As such, presentation and acceptance of gender diversity can vary widely across different international and intranational contexts. Many academic, political, religious, cultural and public health institutions across the world have begun to adapt to this move away from the man/woman gender binary. However, only a few countries have formally acknowledged transgender and gender-diverse individuals within a third gender category, such as by expanding gender-marker options on government-issued identification documents, including in Bangladesh, Canada, Germany, India and Nepal.^11^

There has been a growing commitment in academia, medicine and public health, mostly in higher-income settings, to appreciate and improve the health of transgender and gender-diverse individuals.^11^ Sociocultural experiences of gender identity, however, vary from region to region. As such, medical education, as well as health- and social-care services, must take into consideration the varying cultures and social norms in the local and national contexts across low-, middle- and high-income countries.

### Changes in transition goals

Clinicians must ensure that they are maintaining flexibility in supporting their patient’s transition-related goals. These goals can change and evolve over a person’s life and gender journey. A small minority of individuals who socially or medically transition will have regrets or wish to de-transition. Clinicians may be particularly concerned about these risks, especially if offering medical transition options for a young person since some effects can be irreversible and have long-term impacts on fertility. Several studies found that the rate of regret or wish to de-transition is low, even for adolescents and young people,^27^^,^^29^^,^^33^^–^^35^ who are most likely to be perceived by clinicians as likely to develop regrets.

The Endocrine Society clinical practice guidelines on the treatment of transgender and gender-diverse people recommend the use of gender-affirming hormone therapy in children and adolescents who request this treatment, who have undergone psychiatric assessment and who have maintained a persistent gender identity.^34^ As with all medical interventions, medical transition options require a benefit and risk discussion with the patient. The risks to medical transition, including a small risk of regret or de-transition, have to be balanced with the risk of poor mental health outcomes including suicidality from not permitting access to medical transition for transgender and gender-diverse people.^30^^,^^36^ Rather than a gatekeeper model, clinicians are increasingly advised to approach transition-related care with an informed consent model,^37^ where the risk and likelihood of regret and de-transition are part of the discussion. Puberty suppression is also a frequently used option for adolescents and families who prefer more time to consider the options before starting masculinizing or feminizing hormone therapy.^38^ There is less evidence to guide recommendations around access to transition-related surgeries for adolescents and young people, but some evidence suggests improved mental health outcomes and quality of life and low likelihood of regret after surgery.^27^^,^^29^^,^^34^^,^^35^

### Human rights

In countries where transgender and gender-diverse people are criminalized, the environment around such legislation fosters stigma and discrimination in health-care settings. These legal frameworks impact not only the ability of transgender and gender-diverse people to access health-care services but also the country’s ability to collect comprehensive health-related information. This problem in turn affects the ability of a government to design effective political responses to global and public health concerns. The negative health impact of laws criminalizing transgender and gender-diverse people has been acknowledged and condemned by various international governing bodies.^19^ Countries around the world have committed to upholding the fundamental values enshrined in the Universal Declaration of Human Rights and other treaties and therefore have obligations under international law to protect the human rights of all individuals.^7^^,^^39^^–^^42^ Governments have a duty to review and reform national legislation and policies in line with international human rights standards and the government’s treaty obligations. Dismantling legislation that directly criminalizes transgender and gender-diverse individuals is a fundamental step in supporting them in accessing health and social care services, free from violence and discrimination. Moreover, creating gender-affirming legislation creates an environment where medical schools are able to educate clinicians to provide comprehensive and holistic gender-affirming care. A legal system that prioritizes human rights ensures that all people, including transgender and gender-diverse individuals, are able to enjoy the highest attainable standard of physical and mental health.^19^ Physicians trained to understand the health inequities faced by transgender and gender-diverse people can act as leaders to advocate for needed change.

## Transgender health in medical education

With a few notable exceptions, there is minimal or no inclusion of topics related to transgender health in undergraduate medicine.^43^^–^^49^ Further, in qualitative studies undergraduate educational environments were described by sexual and gender minorities and specifically transgender and gender-diverse medical students as hetero-normative^50^^,^^51^ and cis-normative.^52^ Most transgender and gender-diverse medical students report not disclosing their identities in clinical environments for a variety of reasons, including fear of discrimination and lack of support.^53^ Several recent studies examining the biases held by medical students found evidence of high degrees of bias against individuals not conforming to gender norms.^43^^,^^44^^,^^48^^,^^50^

In terms of postgraduate-level education, most studies consist of survey-level data describing the current state of education programmes, with a few studies exploring the impact of one-time interventions to teach gender-affirming care.^54^^–^^60^ These results demonstrate wide variability of exposure to learning opportunities focused on transgender health, with medical trainees feeling inadequately prepared or lacking in experience to care for transgender and gender-diverse patients.^54^^,^^58^

Several reviews have examined the evidence for educational interventions aimed at increasing medical trainees’ knowledge and confidence in caring for transgender and gender-diverse patients. Two reviews focused on ability to care for sexual and gender minority people, and one review focused solely on transgender and gender-diverse people.^47^^,^^61^^,^^62^ Educational interventions studied included lectures, online modules, panels with sexual and gender minority people speaking about their lived experiences, and encounters with transgender and gender-diverse standardized patients. Overall, the authors noted that while all studied interventions seemed to improve knowledge, confidence or attitudes in post-intervention testing, these findings were limited by the one-time nature of the intervention and the short follow-up time of the post-test.^62^ The studies emphasized the importance of longitudinal curriculum interventions combined with interventions to shift the culture of medical education.^47^^,^^62^ Several studies have noted the value of incorporating concepts of gender-affirming care throughout the curriculum, including highlighting gender diversity as a normal variation of human physiology and experiences.^51^^,^^62^^–^^65^ This competency can be accomplished through didactic approaches and small-group or problem-based learning. Of interest is a recommendation to consider a structural competency framework in teaching issues related to sexual and gender minority health. This approach shifts attention towards factors that influence health outcomes and inequities at levels above individual interactions. Such an initiative would encourage learners to focus not only on the most direct causes of a patient’s presenting issue but also on structural factors which might shape that patient’s lived experiences.^66^

Medical educators have an opportune time to make a concerted effort to provide comprehensive, transgender-specific health-care education for all their trainees,^31^^,^^32^ in line with several global initiatives: the 2016 *Lancet* series on Transgender Health, the World Professional Association for Transgender Health’s *Standards of care for the health of transsexual, transgender, and gender nonconforming people,* version 8 (currently under development), and the publication of the Agenda for Zero Discrimination in Health Care by the Joint United Nations Programme on HIV/AIDS and World Health Organization (WHO).^67^ WHO has also called for medical schools to become more socially accountable by addressing local health inequities. Some medical schools have provided frameworks around teaching diversity and health inequity in medical curricula.^62^^,^^68^ We believe a comprehensive education on transgender health in medical schools requires several key elements: cultural humility and anti-oppression training; involvement of community members; integration of transgender health; practice-focused and in situ training; staff development; and improved access to careers in medicine (Box 1).

Box 1 **Key elements for comprehensive education on transgender health in medical schools**

**Cultural humility and anti-oppression training**

Trainee physicians need a firm grounding in topics such as cultural humility, intersectionality and oppression and how these are related to inequities in health outcomes. Intersectionality describes how the interconnected nature of aspects of one’s identity (such as one’s gender, race and class) leads to different experiences of privilege and discrimination for individuals in a society. Such training is an important precursor to understanding why inequities exist in transgender health.^53^^,^^65^^,^^68^^,^^69^ These topics also provide a foundation for how to address these inequities.

**Involvement of community members**

Transgender health curricula should be developed in consultation with transgender and gender-diverse community members. A participatory action research approach with transgender and gender-diverse community members can help develop and evaluate education initiatives. Community members should be adequately remunerated for their time and involvement.^70^

**Integration of transgender health**

Transgender health should be integrated in a longitudinal manner throughout medical school curricula, rather than being isolated in single lectures on the care of sexual and gender minority people. This training should include material on both transgender-specific health (such as exogenous hormone use) and the care of transgender and gender-diverse people with general health needs (such as the care of a transgender woman presenting with cholecystitis). Every physician will likely have transgender and gender-diverse patients in their practice, regardless of region of residence or specialty.^2^ Physicians therefore need to both be comfortable with gender diversity in their regular practice and have transgender-specific health training.

**Practice-focused and in situ training**

Practice-focused training can be covered through didactic methods, case-based learning or simulated consultations, and should be included in formative and summative testing. Learning objectives for this material have been compiled by organizations including the Association of American Medical Colleges and the Medical Council of Canada.^64^^,^^71^

**Staff development**

A common barrier to teaching transgender health is lack of topic-specific competency among medical school teaching staff.^46^ A successful transgender health curriculum therefore requires training of existing teaching staff in gender diversity and transgender health. This development might involve introducing modules on transgender health into continuing professional development programmes, as well as in postgraduate curricula.^72^^,^^73^

**Improving access to careers in medicine**

Advocating for improved transgender health includes improving access to careers in medicine for transgender and gender-diverse people. For instance, a survey of 1515 Canadian medical students found only 3 (0.2%) students did not identify with the gender on their birth certificate,^74^ lower than the estimated 0.5–1.2% of the population identifying as transgender or gender diverse.^2^ This means that our current classes of medical students do not represent the transgender and gender-diverse population. Data have demonstrated that clinicians who reflect the population they are serving can improve trust in health care and increase access to care and adherence to care recommendations.^75^ For all students, working with diverse student colleagues in medical school is also associated with feeling more prepared to care for patients from backgrounds different from their own.^76^

## Conclusion

While protections for transgender and gender-diverse individuals exist in many global treaties, conventions and international human rights standards, many transgender and gender-diverse individuals are denied their basic human rights including access to adequate health-care services. In many health-care settings, transgender and gender-diverse individuals are subject to stigma and discrimination. Furthermore, data from across the globe demonstrate that physicians and postgraduate and undergraduate medical students are not fully equipped to provide high-quality and comprehensive care to transgender and gender-diverse patients. Integrating holistic approaches to their care in medical school curricula supports the development of health systems that are able to address the needs of these individuals. Including transgender health in medical education is one step towards achieving the 2030 sustainable development agenda of leaving no one behind.
